# Assessing Google Street View Image Availability in Latin American Cities

**DOI:** 10.1007/s11524-019-00408-7

**Published:** 2020-01-03

**Authors:** Dustin Fry, Stephen J. Mooney, Daniel A. Rodríguez, Waleska T. Caiaffa, Gina S. Lovasi

**Affiliations:** 1 Department of Epidemiology and Biostatistics, Drexel University Dornsife School of Public Health, 3600 Market Street 7th Floor, Philadelphia, PA 19104 USA; 2 Department of Epidemiology, University of Washington School of Public Health, 1959 NE Pacific Street, Seattle, WA 98195 USA; 3 Department of City & Regional Planning, University of California–Berkeley College of Environmental Design, 230 Wurster Hall, Berkeley, CA 94720 USA; 4 Department of Preventive and Social Medicine, Federal University of Minas Gerais Observatory for Urban Health in Belo Horizonte, Av. Alfredo Balena, 190, Belo Horizonte, CEP: 30130-100 Brazil

**Keywords:** Latin America, Google Street View, virtual audit, social observation, image availability

## Abstract

Virtual audits using Google Street View are an increasingly popular method of assessing neighborhood environments for health and urban planning research. However, the validity of these studies may be threatened by issues of image availability, image age, and variance of image age, particularly in the Global South. This study identifies patterns of Street View image availability, image age, and image age variance across cities in Latin America and assesses relationships between these measures and measures of resident socioeconomic conditions. Image availability was assessed at 530,308 near-road points within the boundaries of 371 Latin American cities described by the SALURBAL (Salud Urbana en America Latina) project. At the subcity level, mixed-effect linear and logistic models were used to assess relationships between measures of socioeconomic conditions and image availability, average image age, and the standard deviation of image age. Street View imagery was available at 239,394 points (45.1%) of the total sampled, and rates of image availability varied widely between cities and countries. Subcity units with higher scores on measures of socioeconomic conditions had higher rates of image availability (OR = 1.11 per point increase of combined index, *p* < 0.001) and the imagery was newer on average (− 1.15 months per point increase of combined index, *p* < 0.001), but image capture date within these areas varied more (0.59-month increase in standard deviation of image age per point increase of combined index, *p* < 0.001). All three assessed threats to the validity of Street View virtual audit studies spatially covary with measures of socioeconomic conditions in Latin American cities. Researchers should be attentive to these issues when using Street View imagery.

**Electronic supplementary material:**

The online version of this article (10.1007/s11524-019-00408-7) contains supplementary material, which is available to authorized users.

## Introduction

Google Street View, a Google Maps service released in 2007, offers freely available georeferenced panoramic imagery captured at the street level [1]. Since its release, researchers studying the effects of neighborhood environments on health have begun using Street View imagery as a data source. Street View allows the use of a technique called “virtual auditing,” a digital analogue to systematic social observation. Trained auditors “travel” through neighborhoods within the Street View interface and characterize the environment based on the study’s variables of interest. A recent systematic review identified at least 54 studies using virtual audits in health research, most of which rely on Google Street View imagery [2].

Studies that compare data obtained from virtual audits in Google Street View to data from in-person audits generally find good concordance between the two techniques [3–8], although virtual auditing does not seem to work well for certain instruments [9, 10]. Researchers note that image resolution can limit the reliable identification of small objects, and that the inability of researchers to control when images are taken limits Street View’s usefulness in assessing variables that can change rapidly over time [3–10]. This suggests that virtual audits are not a good substitution for in-person audits in all cases.

Aside from these limitations, some of the main concerns about the feasibility and validity of using Street View imagery in health research involve image availability and frequency of update. Lack of imagery is an important reason that few virtual audits have been performed in low- or middle-income countries [2], and can limit research in two ways. If imagery is very sparse or completely unavailable in a study area, virtual audits cannot be used at all. If some but not all streets are imaged, and streets with available imagery are different on average than streets without imagery (for example, if streets in more popularly visited, densely populated, or wealthier areas are more likely to be imaged), studies could suffer from non-ignorable missing data [11]. Informal communities may be particularly affected by issues of image availability: informal communities may lack passable roads, or the roads and paths that are present may not be mapped, with either situation precluding Google Street View imagery of the area.

Further, Street View imagery is not updated at consistent intervals, meaning that some areas have newer imagery available than other areas [2]. Because a static image represents one point in time, virtual audits act only as a direct measure of neighborhood conditions at the time of imagery capture [3]. Even within small-scale areas of cities, image age can vary between streets, often changing after an intersection [12]. Imagery dates that change across a study area can make virtual audits challenging as they limit researchers’ ability to assess a particular street at a particular point in time. For virtual audits to most accurately measure neighborhood conditions, a study area would have Street View imagery that is [1] universally available, [2] captured close to the time point of interest, and [3] captured at approximately the same point in time throughout the area of interest. Spatial covariance between image availability or update frequency and measures of socioeconomic conditions could pose a threat to the validity of virtual audit studies in Latin America.

This study seeks to better understand these issues of image availability and update frequency in the context of Latin American cities. The SALURBAL (Salud Urbana en America Latina) project has consolidated demographic and economic data from cities across Central and South America [13]. We used these data and the Google Street View Application Programming Interface (API) to describe variation in image availability, image age, and variance of image age across 371 Latin American cities, and to quantify associations between these variables with measures of socioeconomic conditions.

## Methods

### SALURBAL Cities and Subcity Units

The SALURBAL data platform has been described in detail elsewhere [13]. Briefly, all cities with population ≥ 100,000 in Argentina, Brazil, Chile, Colombia, Costa Rica, El Salvador, Guatemala, Mexico, Nicaragua, Panamá, and Perú were identified. “Level 1 Administrative” city boundaries were delineated based on clusters of administratively defined urban areas. Alternative city boundaries have also been identified based on government-defined metropolitan areas or based on satellite imagery of the extent of the built-up environment. “Level 2” subcity boundaries were identified based on the smallest geographic unit with readily available linked data sources (such as mortality records). In total, 1436 subcity units within 371 cities were characterized.

At the subcity level, census and survey data have been compiled and harmonized. Independent variables in this analysis included population density, the proportion of households living above the poverty line, the proportion of dwellings with exterior walls mostly composed of durable materials, the proportion of households with piped water inside the dwelling, the proportion of households connected to a public sewage network, the proportion of the population aged 25 or over who completed secondary education or above (12 years of education), and labor force participation.

The outcomes of interest (image availability, image age, and variance of image age) were assessed by sampling points spatially, identifying the sampled points near road networks, and then assessing the most recent available image adjacent to each of the near-road points, as is described in further detail below.

### Assessing Image Availability and Age

**Fig. 1:**
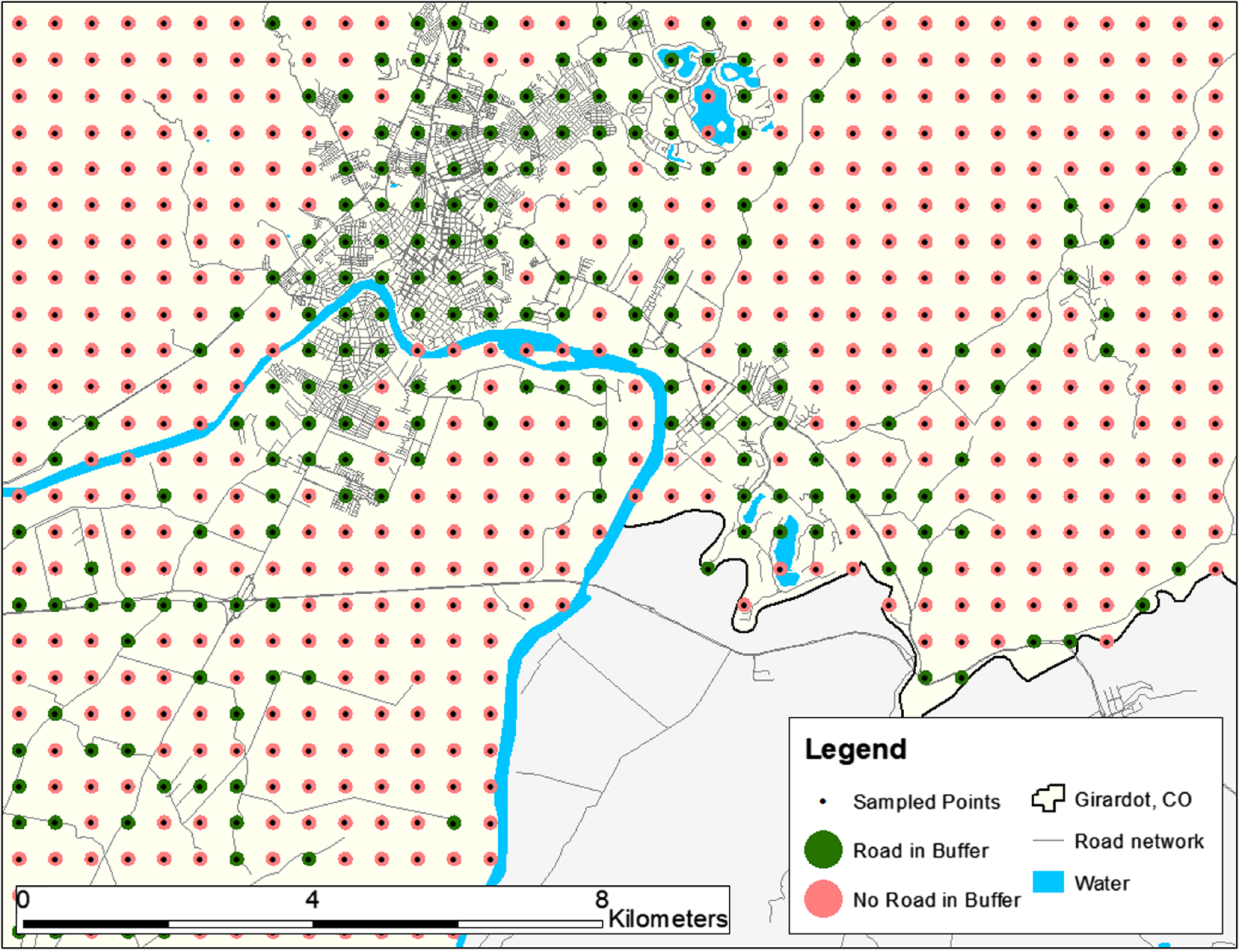
Illustration of how points were sampled in a section of the level 1 administrative area of Girardot, Colombia. Points without a road within 100 m were excluded from analysis. Water area and road network data from Open Street Maps

Using the Level 1 Administrative city boundaries from SALURBAL, points were placed at 500-m intervals across the entire area of all 371 cities in the sample for a total of 5,090,496 points. One hundred–meter buffers were constructed around each point and the total length of road network from Open Street Maps data [14] (accessed 9 April 2019) within each buffer was calculated. Points with no road within the buffer were discarded for a final sample of 530,308 eligible points, as illustrated in Fig. 1. Spatial analysis was conducted in ArcMap 10.5 [15].

Using the Python client for the Google Street View API [16], metadata requests were made for every point in the sample to assess the availability of any Street View imagery within a 100-m radius of each point. If available, the image date (month and year) of the closest image to each point was also captured; if imagery existed at multiple time points, the most recent image was captured. The metadata requests were made between 15 April 2019 and 17 April 2019. A total of 239,394 points (45.1%) returned available imagery, 290,886 (54.9%) did not, and 28 requests (0.005%) returned with an error; because these errors appear to be randomly spatially distributed, these data were assumed to be missing completely at random and complete case analysis was used.

Image age was assessed at the subcity level, operationalized as the mean number of months prior to April 2019 that the imagery in each subcity unit was taken. Variance of image age was also assessed at the subcity level, operationalized as the standard deviation of the age of available imagery in months.

Although it would have been ideal to use the Google Maps road layer in all analyses, this is not possible because Google’s proprietary data is not downloadable to other GIS systems. Therefore, to validate the assumption that the points excluded due to a lack of adjacent road would not have Google Street View images available, metadata requests for a simple random sample of 10,000 of the excluded points were made on 28 May 2019. Out of these 10,000, 30 points (0.3%) returned available imagery. These seem due to minor differences between the Open Street Maps road network and the Google Maps road network and appear to be randomly distributed across the study cities. The opposite situation is also possible: due to the same differences between maps, we may be failing to exclude points for which there is no corresponding road in Google Maps, although we expect that the magnitude of this problem is similarly small and similarly randomly distributed across cities.

### Statistical Analysis

To analyze the relationships between social and built-environmental variables and image availability, mixed-effect logistic regression models were computed for each variable of interest. The binary availability of any Street View imagery within a 100-m radius of each sampled point was predicted in separate models by the population density, the proportion of households with piped water, the proportion of households connected to the sewer, the proportion of households with durable walls, the proportion of residents participating in the labor force, the proportion of residents above age 25 who completed secondary education or above, and the proportion of households living above the poverty line at the subcity level. Each model controlled for the length of the road network within a 100-m radius of each sampled point, country, and the area of the L2 subcity area as fixed effects. To account for spatial autocorrelation of image availability across cities, each model also included a random slope spatially across the city, fitted via *X* and *Y* coordinates that were *Z*-score standardized to the center of mass of each city’s sampled points. The Central American countries (El Salvador, Nicaragua, Panamá, Costa Rica, and Guatemala) were pooled due to a low number of cities in each country. Each variable of interest and the length of the road network were standardized by a *Z*-score transformation such that odds ratios can be interpreted per standard deviation of the independent variable.

Models with image age or image age variance as the outcome used mixed-effect linear regression, the unit of analysis was the subcity unit rather than the individual point, and each city therefore had a random intercept rather than a random slope across space. The modeling approach was otherwise parallel to the analyses of image availability, so coefficients can be interpreted as a difference in expected value of the outcome per standard deviation of each independent variable.

Finally, to assess the associations of image availability with the variables of interest in combination, the *Z*-score of the proportion of households living above the poverty line, the proportion of dwellings with exterior walls mostly composed of durable materials, the proportion of households with piped water inside the dwelling, the proportion of households connected to a public sewage network, the proportion of the population aged 25 or over who completed secondary education or above, and labor force participation were summed at the subcity level to compute an index of socioeconomic conditions. Because data on poverty were not available at the subcity level in Argentina and not available at all in Costa Rica or Nicaragua, two indices were computed: one with poverty included and one without (correlation where both could be calculated = 0.98). The index without poverty rate as an included variable ranged from − 11.9 to 7.27 and the index including poverty rate ranged from − 12.03 to 8.9; in both indices, higher scores indicate greater socioeconomic conditions at the subcity level. The same modeling approach as described above was used to assess relationships between these indices and image availability, image age, and standard deviation of image age, with the exception that the index was not transformed.

All statistical analysis was conducted in R 3.5.1 [17]. Multilevel models were computed using the lme4 package [18].^(p4)^

## Results

Results at the country level are shown in Table 1. Image availability ranged from a minimum of 8.3% of sampled points in Central America to a maximum of 52.2% of sampled points in Mexico. Image age ranged from a minimum of 27.2 months in Central America to a maximum of 65.4 months in Chile, while the standard deviation of image age ranged from a minimum of 10.6 months in Perú to a maximum of 31.9 months in Brazil.

**Table 1:** Results at the country level. *N* for each country is the number of sampled points. Availability is expressed as the percentage of sampled points in each country that returned available imagery. Age is expressed as the mean age of available imagery in each country in months as of April 2019. Age variability is expressed as the standard deviation of image age in each country

Country	*n*	Availability (%)	Age (months)	Age variability (months)
Argentina	81,101	35.7	51.5	15.2
Brazil	224,313	47.6	59.1	31.9
Central America	17,002	8.3	27.2	11
Chile	27,131	39.6	65.4	16.3
Colombia	27,736	45.5	51.2	22.2
Mexico	135,191	52.2	55.9	32.6
Peru	17,834	46.6	59.7	10.6

At the city level, image availability ranged from a minimum of 0.8% of sampled points in Chinandega, Nicaragua, to a maximum of 87.3% of sampled points in Iraputo, Mexico, with a median of 43.3%. Mean image age ranged from a minimum of 9.3 months in San Miguel, El Salvador, to a maximum of 116.7 months (nearly 10 years) in Matamoros, Mexico, with a median of 61.4 months. The standard deviation of image age ranged from a minimum of 2.4 months in Macapá, Brazil, to a maximum of 55.6 months in Ciudad Juárez, Mexico, with a median of 21.0 months.

**Fig. 2:**
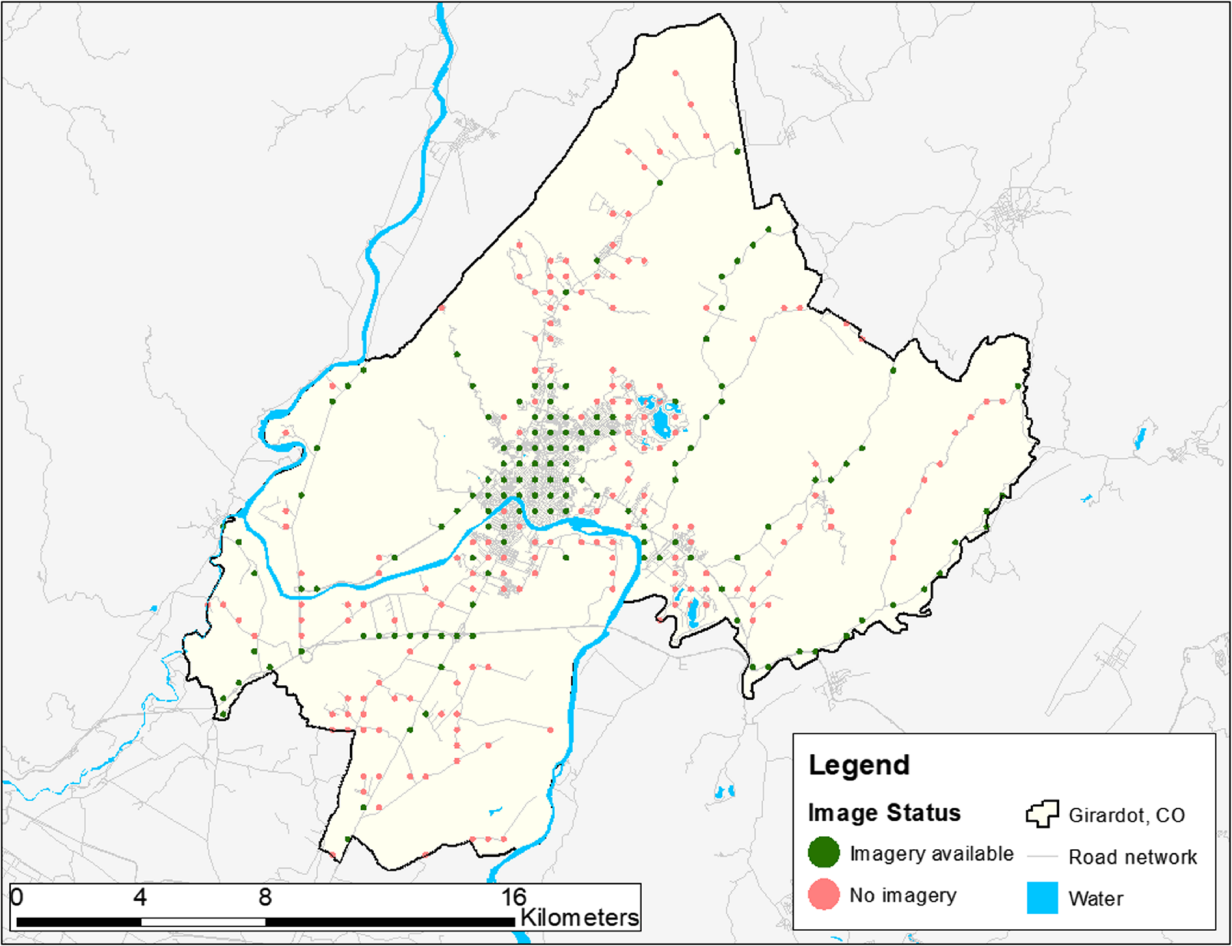
Patterns of image availability for the level 1 administrative area of Girardot, Colombia. Red and green points have a 100-m radius. Population, 139,155. Sampled points, 314. Image availability, 43.3%. Water area and road network data from Open Street Maps

Figure 2 displays the spatial patterns of image availability for Girardot, Colombia, which was selected as a representative city because it had the median level of image availability across all cities.

### Image Availability

Results for the image availability models are shown in the first column of Table 2. Controlling for the length of the road network around each point, the size of the subcity unit, and country as fixed effects and with a random slope geographically across each city, population density was positively associated with the odds of image availability, as were the proportion of households with piped water, the proportion of households connected to the sewer, the proportion of households with durable walls, the labor force participation rate, the proportion of residents with a secondary education or above, and the proportion of households above the poverty line. Both of the combined index models (with and without the proportion of households above the poverty line as an included variable) indicated a positive relationship between socioeconomic conditions and image availability. In summary, subcity units with higher measures of socioeconomic conditions tended to have greater levels of image availability on average.

**Table 2:** Each variable of interest is modeled in separate mixed logistic regression models (image availability models) or linear mixed effects regressions (image age and image age variability) with fixed effects for country and the area of the L2 subcity unit and either a variability). Image availability models have an additional fixed effect for the length of road network surrounding each individual point random slope geographically across each city (image availability models) or a random intercept for city (image age and image age

	Image Availability					Image Age^†^				Image Age Variability^†^			
Variable	*n*	*b*	OR	SE	*p*	*n*	*b*	SE	*p*	*n*	*b*	SE	*p*
Population density*	530,280	0.03	1.03	0.005	<0.001	1,370	0.30	0.335	0.376	1,370	-0.53	0.194	0.006
% water in household*	530,238	0.15	1.16	0.008	<0.001	1,369	-2.67	0.455	<0.001	1,369	1.58	0.263	<0.001
% household connected to sewer*	530,238	0.27	1.32	0.009	<0.001	1,369	-3.04	0.413	<0.001	1,369	1.57	0.239	<0.001
% household has durable walls*	530,238	0.41	1.51	0.016	<0.001	1,369	-2.49	0.523	<0.001	1,369	1.25	0.296	<0.001
% labor participation*	530,238	0.34	1.41	0.012	<0.001	1,369	-3.42	0.592	<0.001	1,369	1.89	0.342	<0.001
% secondary education*	530,238	0.23	1.25	0.006	<0.001	1,369	-4.27	0.435	<0.001	1,369	1.91	0.258	<0.001
% above poverty line*	424,146	0.33	1.39	0.013	<0.001	1,154	-4.57	0.687	<0.001	1,154	2.57	0.393	<0.001
Combined index without poverty	530,238	0.11	1.11	0.003	<0.001	1,369	-1.15	0.126	<0.001	1,369	0.59	0.074	<0.001
Combined index with poverty	424,146	0.09	1.10	0.003	<0.001	1,154	-0.99	0.123	<0.001	1,154	0.54	0.072	<0.001

*Variables are *Z*-score standardized, so odds ratios and linear parameters are interpretable per standard deviation change in the independent variable

^†^Models are at the level of the L2 subcity unit, not at the level of the individual sampled point

### Image Age

Results for the image age models are shown in the second column of Table 2. Controlling for country and the size of the subcity unit as fixed effects and with a random intercept for city, there was no significant association between population density and average image age at the subcity level. The proportion of households with piped water, the proportion of households connected to the sewer, the proportion of households with durable walls, the labor force participation rate, the proportion of residents with a secondary education or above, and the proportion of households above the poverty line were all negatively associated with average image age at the subcity level. Both of the combined index models (with and without the proportion of households above the poverty line as an included variable) indicated a negative relationship between socioeconomic conditions and average image age at the subcity level. In summary, subcity units with higher measures of socioeconomic conditions tended to have newer available imagery on average.

### Image Age Variance

Results for the temporal stability models are shown in the third column of Table 2. Controlling for country and the size of the subcity unit as fixed effects and with a random intercept for city, population density was negatively associated with the standard deviation of image age at the subcity level. The proportion of households with piped water, the proportion of households connected to the sewer, the proportion of households with durable walls, the labor force participation rate, the proportion of residents with a secondary education or above, and the proportion of households above the poverty line were all positively associated with the standard deviation of image age at the subcity level. Both of the combined index models (with and without the proportion of households above the poverty line as an included variable) indicated a positive relationship between socioeconomic conditions and the standard deviation of image age at the subcity level. In summary, subcity units with higher measures of socioeconomic conditions tended to have a greater level of variance of image capture dates.

Modeling results for image availability, image age, and image age variance are also presented as stratified by country in supplementary tables.

## Discussion

This evaluation of Google Street View image availability and update frequency in Latin America provides evidence that all three assessed threats to the validity of virtual audits of neighborhood environments (lack of image availability, older image age, and variance of image age) spatially covary with measures of socioeconomic conditions. In subcity units with high measures of socioeconomic conditions, Street View imagery is more consistently available and tends to be newer. Conversely, however, image capture dates vary less in subcity units with lower measures of socioeconomic conditions. Researchers seeking to use Google Street View to evaluate neighborhood environments in Latin American cities should be attentive to these issues, particularly the risk that neighborhood measures could be compromised by differential rates of missing data.

Previous work has highlighted image availability as a specific limitation of virtual audits [2] and explored variance in image age over small areas of cities [12], but to our knowledge this is the first study to examine such issues in the Latin American context and the first to assess associations between image availability and other contextual variables. Prior to conducting virtual audit studies, researchers should first carefully evaluate the Street View imagery that is available in the study site, including the spatial patterns of availability and the dates of image capture. If areas of researcher interest have high rates of non-imaged street segments, or if the imagery is not available at time points of interest, measures from virtual audits could be supplemented by other data sources such as in-person audits or ecometric resident surveys [19, 20]. Readily available sources of external data include the SALURBAL-compiled data used for this analysis and survey data from the Development Bank of Latin America [21].

In places with low rates of image availability, virtual audits should be used with caution, as comparisons across and within cities may be biased by omission of the most vulnerable areas. The effect of this issue may be even greater in practice than was found in this analysis: because informal communities may not have mapped roads or paths in places where people live, there may be no potential for Street View imagery to be present. Further, we considered only a binary of image availability as contrasted from image age. In practice, imagery that is deemed by researchers to be too distant from the time point of interest may as well be considered unavailable, and because these two dimensions of image availability are both found in the same areas of cities, they may act in concert to reduce the feasibility of virtual audit studies.

Based on an assessment of 1436 subcity units across 371 cities, this study provides a broad summary of image availability and update frequency in Latin America. However, several limitations should be noted. First, because of limits to the spatial resolution of available socioeconomic data across all cities, this study should be considered as an ecological assessment; the mean area of the subcity units analyzed here is 890 km^2^ and ranges between 0.3 and 53,302 km^2^. Caution should therefore be taken in interpreting our results, as they apply to differences between subcity units and not to differences between individual streets; it should not be inferred that the same relationships apply within subcity units as between subcity units. Second, the socioeconomic variables used in this analysis were not gathered at the same time across all variables or all countries, with collection dates ranging between 2002 and 2016. While this temporal mismatch is not ideal, neighborhood socioeconomic conditions change slowly, and so differences are unlikely to qualitatively invalidate our findings. Third, we selected the closest image to the road point sampled, although other images within the 100-m buffer may have different age and quality; researchers may be able to deliberately select imagery that would be most representative of the neighborhood environment. Fourth, the analysis is restricted to areas with mapped roads to assess Street View imagery once areas have been mapped; an analysis of the presence or absence of mapped roads is beyond the scope of this work, although as noted, this is an important determinant of the validity of virtual audit studies. Finally, the fact that our analysis combines data across so many areas limits its ability to describe any one city; we are seeking to provide an overview of broad patterns of image availability and age rather than to gain a detailed understanding of a particular city of interest.

At the time of writing, Google appears to remain committed to maintaining and improving Google Street View as a service by imaging additional streets and updating existing imagery [22]. As a result, the relationships described here are not expected to be static; as the proportion of imaged streets in Latin America approaches 100%, the potential for a meaningful difference in availability between neighborhoods diminishes. However, for the time being, the differential rates of availability between neighborhoods based on socioeconomic characteristics pose a potential challenge to the validity of studies conducted using virtual audits in Latin American cities.

## Electronic supplementary material

ESM 1 (DOCX 41 kb)
