# Supplementary material for: Assessing Google Street View Image Availability in Latin American Cities
Source: J Urban Health. 2020 Jan 3;97(4):552–60. doi: 10.1007/s11524-019-00408-7 (PMC7392983; doi:10.1007/s11524-019-00408-7)
Supplement: Supplementary file 1 — (DOCX 41 kb) [file 11524_2019_408_MOESM1_ESM.docx]

Supplementary Table 1: Results for Argentina

|  | **Image Availability** | | | | | **Image Age** | | | | **Image Age Variability** | | | |
| --- | --- | --- | --- | --- | --- | --- | --- | --- | --- | --- | --- | --- | --- |
| **Variable** | **n** | ***b*** | **OR** | **SE** | **p** | **n** | ***b*** | **SE** | **p** | **n** | ***b*** | **SE** | **p** |
| Population density | 81100 | 0.26 | 1.29 | 0.015 | <0.001 | 109 | -7.10 | 0.573 | <0.001 | 109 | -1.39 | 0.450 | 0.003 |
| % water in household | 81100 | 0.17 | 1.19 | 0.016 | <0.001 | 109 | -5.20 | 0.715 | <0.001 | 109 | 1.78 | 0.441 | <0.001 |
| % household connected to sewer | 81100 | 0.17 | 1.18 | 0.017 | <0.001 | 109 | -6.43 | 0.594 | <0.001 | 109 | 0.70 | 0.460 | 0.131 |
| % household has durable walls | 81100 | 0.70 | 2.02 | 0.046 | <0.001 | 109 | -3.39 | 0.849 | <0.001 | 109 | 0.34 | 0.505 | 0.499 |
| % labor participation | 81100 | 0.15 | 1.16 | 0.018 | <0.001 | 109 | -5.68 | 0.749 | <0.001 | 109 | 1.47 | 0.487 | 0.003 |
| % secondary education | 81100 | 0.08 | 1.09 | 0.012 | <0.001 | 109 | -6.50 | 0.524 | <0.001 | 109 | 0.54 | 0.437 | 0.222 |
| % above poverty line |  |  |  |  |  |  |  |  |  |  |  |  |  |
| Combined index without poverty | 81100 | 0.10 | 1.11 | 0.009 | <0.001 | 109 | -2.80 | 0.204 | <0.001 | 109 | 0.40 | 0.180 | 0.028 |
| Combined index with poverty |  |  |  |  |  |  |  |  |  |  |  |  |  |

Supplementary Table 2: Results for Brasil

|  | **Image Availability** | | | | | **Image Age** | | | | **Image Age Variability** | | | |
| --- | --- | --- | --- | --- | --- | --- | --- | --- | --- | --- | --- | --- | --- |
| **Variable** | **n** | ***b*** | **OR** | **SE** | **p** | **n** | ***b*** | **SE** | **p** | **n** | ***b*** | **SE** | **p** |
| Population density | 224,303 | 0.19 | 1.21 | 0.011 | <0.001 | 422 | -3.49 | 0.748 | <0.001 | 422 | -0.49 | 0.415 | 0.241 |
| % water in household | 224,303 | 0.22 | 1.25 | 0.017 | <0.001 | 422 | -2.06 | 0.840 | 0.015 | 422 | 2.21 | 0.436 | <0.001 |
| % household connected to sewer | 224,303 | 0.37 | 1.45 | 0.014 | <0.001 | 422 | -3.64 | 0.816 | 0.000 | 422 | 2.55 | 0.425 | <0.001 |
| % household has durable walls | 224,303 | 0.70 | 2.02 | 0.029 | <0.001 | 422 | -2.37 | 0.987 | 0.017 | 422 | 2.11 | 0.513 | <0.001 |
| % labor participation | 224,303 | 0.21 | 1.24 | 0.013 | <0.001 | 422 | -1.93 | 0.800 | 0.016 | 422 | 0.88 | 0.431 | 0.041 |
| % secondary education | 224,303 | 0.26 | 1.30 | 0.009 | <0.001 | 422 | -4.34 | 0.663 | <0.001 | 422 | 2.38 | 0.365 | <0.001 |
| % above poverty line | 224,303 | 0.40 | 1.49 | 0.020 | <0.001 | 422 | -2.64 | 0.918 | 0.004 | 422 | 2.34 | 0.467 | <0.001 |
| Combined index without poverty | 224,303 | 0.20 | 1.22 | 0.006 | <0.001 | 422 | -2.06 | 0.354 | <0.001 | 422 | 1.40 | 0.183 | <0.001 |
| Combined index with poverty | 224,303 | 0.16 | 1.18 | 0.005 | <0.001 | 422 | -1.69 | 0.299 | <0.001 | 422 | 1.20 | 0.153 | <0.001 |

Supplementary Table 3: Results for Central America

|  | **Image Availability** | | | | | **Image Age** | | | | **Image Age Variability** | | | |
| --- | --- | --- | --- | --- | --- | --- | --- | --- | --- | --- | --- | --- | --- |
| **Variable** | **n** | ***b*** | **OR** | **SE** | **p** | **n** | ***b*** | **SE** | **p** | **n** | ***b*** | **SE** | **p** |
| Population density | 17,001 | -0.38 | 0.69 | 0.049 | <0.001 | 100 | 0.57 | 1.002 | 0.572 | 100 | -2.37 | 0.733 | 0.002 |
| % water in household | 17,001 | 0.74 | 2.09 | 0.079 | <0.001 | 100 | 0.34 | 1.225 | 0.783 | 100 | 2.15 | 0.794 | 0.008 |
| % household connected to sewer | 17,001 | 0.54 | 1.71 | 0.042 | <0.001 | 100 | 0.79 | 1.006 | 0.432 | 100 | 0.56 | 0.821 | 0.497 |
| % household has durable walls | 17,001 | 0.31 | 1.37 | 0.091 | 0.001 | 100 | -0.56 | 1.184 | 0.640 | 100 | -0.63 | 0.918 | 0.494 |
| % labor participation | 17,001 | 0.63 | 1.88 | 0.053 | <0.001 | 100 | -0.31 | 1.083 | 0.777 | 100 | 1.39 | 0.840 | 0.105 |
| % secondary education | 17,001 | 0.77 | 2.17 | 0.057 | <0.001 | 100 | -1.83 | 1.013 | 0.087 | 100 | 1.87 | 0.815 | 0.027 |
| % above poverty line | 9,818 | 0.63 | 1.87 | 0.055 | <0.001 | 62 | -0.80 | 1.489 | 0.592 | 62 | 2.21 | 1.023 | 0.035 |
| Combined index without poverty | 17,001 | 0.21 | 1.23 | 0.016 | <0.001 | 100 | -0.08 | 0.317 | 0.794 | 100 | 0.46 | 0.247 | 0.068 |
| Combined index with poverty | 9,818 | 0.19 | 1.21 | 0.015 | <0.001 | 62 | 0.00 | 0.358 | 0.996 | 62 | 0.46 | 0.242 | 0.059 |

Supplementary Table 4: Results for Chile

|  | **Image Availability** | | | | | **Image Age** | | | | **Image Age Variability** | | | |
| --- | --- | --- | --- | --- | --- | --- | --- | --- | --- | --- | --- | --- | --- |
| **Variable** | **n** | ***b*** | **OR** | **SE** | **p** | **n** | ***b*** | **SE** | **p** | **n** | ***b*** | **SE** | **p** |
| Population density | 27,127 | 0.19 | 1.21 | 0.031 | <0.001 | 81 | 0.40 | 0.719 | 0.582 | 81 | 0.58 | 0.503 | 0.253 |
| % water in household | 27,127 | 0.15 | 1.16 | 0.031 | <0.001 | 81 | -0.02 | 0.782 | 0.983 | 81 | 0.96 | 0.528 | 0.074 |
| % household connected to sewer | 27,127 | 0.11 | 1.12 | 0.030 | <0.001 | 81 | 0.03 | 0.779 | 0.964 | 81 | 0.67 | 0.537 | 0.218 |
| % household has durable walls | 27,127 | 0.27 | 1.31 | 0.065 | <0.001 | 81 | -0.19 | 0.860 | 0.826 | 81 | 1.10 | 0.557 | 0.056 |
| % labor participation | 27,127 | 0.14 | 1.15 | 0.032 | <0.001 | 81 | -2.07 | 0.706 | 0.004 | 81 | 0.29 | 0.526 | 0.583 |
| % secondary education | 27,127 | 0.12 | 1.13 | 0.025 | <0.001 | 81 | -1.44 | 0.629 | 0.025 | 81 | 0.15 | 0.470 | 0.755 |
| % above poverty line | 27,127 | -0.12 | 0.88 | 0.047 | 0.008 | 81 | -1.38 | 0.793 | 0.086 | 81 | 0.39 | 0.566 | 0.488 |
| Combined index without poverty | 27,127 | 0.07 | 1.07 | 0.013 | <0.001 | 81 | -0.38 | 0.247 | 0.132 | 81 | 0.27 | 0.171 | 0.124 |
| Combined index with poverty | 27,127 | 0.06 | 1.07 | 0.013 | <0.001 | 81 | -0.37 | 0.236 | 0.120 | 81 | 0.25 | 0.163 | 0.131 |

Supplementary Table 5: Results for Colombia

|  | **Image Availability** | | | | | **Image Age** | | | | **Image Age Variability** | | | |
| --- | --- | --- | --- | --- | --- | --- | --- | --- | --- | --- | --- | --- | --- |
| **Variable** | **n** | ***b*** | **OR** | **SE** | **p** | **n** | ***b*** | **SE** | **p** | **n** | ***b*** | **SE** | **p** |
| Population density | 27,736 | -0.07 | 0.94 | 0.030 | 0.029 | 84 | 1.27 | 1.408 | 0.369 | 84 | -2.38 | 0.639 | <0.001 |
| % water in household | 27,736 | -0.14 | 0.87 | 0.042 | 0.001 | 84 | 0.52 | 1.423 | 0.714 | 84 | 1.86 | 0.654 | 0.006 |
| % household connected to sewer | 27,736 | -0.03 | 0.98 | 0.035 | 0.478 | 84 | -0.33 | 1.358 | 0.809 | 84 | 1.85 | 0.606 | 0.003 |
| % household has durable walls | 27,736 | 0.31 | 1.36 | 0.078 | <0.001 | 84 | -0.43 | 1.961 | 0.827 | 84 | 1.63 | 0.930 | 0.085 |
| % labor participation | 27,736 | 0.11 | 1.12 | 0.029 | <0.001 | 84 | -1.43 | 1.375 | 0.301 | 84 | 1.97 | 0.603 | 0.002 |
| % secondary education | 27,736 | 0.09 | 1.10 | 0.025 | <0.001 | 84 | 0.68 | 1.266 | 0.593 | 84 | 2.44 | 0.481 | <0.001 |
| % above poverty line | 9,927 | 0.21 | 1.23 | 0.212 | 0.327 |  |  |  |  |  |  |  |  |
| Combined index without poverty | 27,736 | 0.02 | 1.02 | 0.015 | 0.095 | 84 | -0.07 | 0.497 | 0.880 | 84 | 0.83 | 0.211 | <0.001 |
| Combined index with poverty | 9,927 | 0.05 | 1.05 | 0.087 | 0.591 |  |  |  |  |  |  |  |  |

Supplementary Table 6: Results for Mexico

|  | **Image Availability** | | | | | **Image Age** | | | | **Image Age Variability** | | | |
| --- | --- | --- | --- | --- | --- | --- | --- | --- | --- | --- | --- | --- | --- |
| **Variable** | **n** | ***b*** | **OR** | **SE** | **p** | **n** | ***b*** | **SE** | **p** | **n** | ***b*** | **SE** | **p** |
| Population density | 135,179 | 0.03 | 1.03 | 0.008 | <0.001 | 406 | 1.28 | 0.653 | 0.051 | 406 | -0.73 | 0.385 | 0.059 |
| % water in household | 135,179 | 0.19 | 1.21 | 0.013 | <0.001 | 406 | -3.86 | 0.784 | <0.001 | 406 | 1.20 | 0.464 | 0.010 |
| % household connected to sewer | 135,179 | 0.23 | 1.26 | 0.015 | <0.001 | 406 | -2.88 | 0.800 | <0.001 | 406 | 0.78 | 0.468 | 0.096 |
| % household has durable walls | 135,179 | 0.27 | 1.31 | 0.030 | <0.001 | 406 | -4.59 | 1.105 | <0.001 | 406 | 1.55 | 0.622 | 0.013 |
| % labor participation | 135,179 | 0.24 | 1.27 | 0.012 | <0.001 | 406 | -3.06 | 0.736 | <0.001 | 406 | 1.42 | 0.432 | 0.001 |
| % secondary education | 135,179 | 0.19 | 1.21 | 0.010 | <0.001 | 406 | -3.84 | 0.668 | <0.001 | 406 | 1.54 | 0.403 | <0.001 |
| % above poverty line | 135,179 | 0.20 | 1.23 | 0.013 | <0.001 | 406 | -4.24 | 0.857 | <0.001 | 406 | 1.45 | 0.505 | 0.004 |
| Combined index without poverty | 135,179 | 0.09 | 1.09 | 0.005 | <0.001 | 406 | -1.49 | 0.258 | <0.001 | 406 | 0.54 | 0.155 | 0.001 |
| Combined index with poverty | 135,179 | 0.07 | 1.08 | 0.004 | <0.001 | 406 | -1.28 | 0.219 | <0.001 | 406 | 0.46 | 0.132 | 0.001 |

Supplementary Table 7: Results for Peru

|  | **Image Availability** | | | | | **Image Age** | | | | **Image Age Variability** | | | |
| --- | --- | --- | --- | --- | --- | --- | --- | --- | --- | --- | --- | --- | --- |
| **Variable** | **n** | ***b*** | **OR** | **SE** | **p** | **n** | ***b*** | **SE** | **p** | **n** | ***b*** | **SE** | **p** |
| Population density | 17,834 | -0.13 | 0.88 | 0.032 | <0.001 | 168 | 0.20 | 0.446 | 0.653 | 168 | -0.07 | 0.324 | 0.820 |
| % water in household | 17,792 | 0.17 | 1.18 | 0.026 | <0.001 | 167 | -1.56 | 0.430 | <0.001 | 167 | 0.85 | 0.318 | 0.008 |
| % household connected to sewer | 17,792 | 0.17 | 1.19 | 0.025 | <0.001 | 167 | -1.59 | 0.450 | 0.001 | 167 | 0.92 | 0.330 | 0.006 |
| % household has durable walls | 17,792 | 0.22 | 1.24 | 0.044 | <0.001 | 167 | -1.79 | 0.510 | 0.001 | 167 | 0.37 | 0.369 | 0.312 |
| % labor participation | 17,792 | 0.35 | 1.42 | 0.041 | <0.001 | 167 | -1.62 | 0.523 | 0.002 | 167 | 0.53 | 0.379 | 0.162 |
| % secondary education | 17,792 | 0.28 | 1.32 | 0.033 | <0.001 | 167 | -2.16 | 0.496 | <0.001 | 167 | 0.97 | 0.365 | 0.009 |
| % above poverty line | 17,792 | 0.26 | 1.30 | 0.036 | <0.001 | 167 | -1.81 | 0.501 | <0.001 | 167 | 0.91 | 0.354 | 0.011 |
| Combined index without poverty | 17,792 | 0.06 | 1.06 | 0.007 | <0.001 | 167 | -0.52 | 0.112 | <0.001 | 167 | 0.22 | 0.084 | 0.009 |
| Combined index with poverty | 17,792 | 0.06 | 1.06 | 0.007 | <0.001 | 167 | -0.50 | 0.102 | <0.001 | 167 | 0.22 | 0.077 | 0.005 |
